# Cyclophilin C Participates in the US2-Mediated Degradation of Major Histocompatibility Complex Class I Molecules

**DOI:** 10.1371/journal.pone.0145458

**Published:** 2015-12-21

**Authors:** Daniel C. Chapman, Pawel Stocki, David B. Williams

**Affiliations:** 1 Department of Immunology, University of Toronto, Toronto, Ontario, Canada; 2 Department of Biochemistry, University of Toronto, Toronto, Ontario, Canada; University of London, St George's, UNITED KINGDOM

## Abstract

Human cytomegalovirus uses a variety of mechanisms to evade immune recognition through major histocompatibility complex class I molecules. One mechanism mediated by the immunoevasin protein US2 causes rapid disposal of newly synthesized class I molecules by the endoplasmic reticulum-associated degradation pathway. Although several components of this degradation pathway have been identified, there are still questions concerning how US2 targets class I molecules for degradation. In this study we identify cyclophilin C, a peptidyl prolyl isomerase of the endoplasmic reticulum, as a component of US2-mediated immune evasion. Cyclophilin C could be co-isolated with US2 and with the class I molecule HLA-A2. Furthermore, it was required at a particular expression level since depletion or overexpression of cyclophilin C impaired the degradation of class I molecules. To better characterize the involvement of cyclophilin C in class I degradation, we used LC-MS/MS to detect US2-interacting proteins that were influenced by cyclophilin C expression levels. We identified malectin, PDIA6, and TMEM33 as proteins that increased in association with US2 upon cyclophilin C knockdown. In subsequent validation all were shown to play a functional role in US2 degradation of class I molecules. This was specific to US2 rather than general ER-associated degradation since depletion of these proteins did not impede the degradation of a misfolded substrate, the null Hong Kong variant of α_1_-antitrypsin.

## Introduction

Major histocompatibility complex class I molecules (MHC class I, MHC I) play a crucial role in cellular immunity by presenting antigenic peptides to CD8^+^ T cells. They are heterotrimers consisting of a membrane-anchored heavy chain (HC), a soluble subunit termed β_2_-microglobulin (β_2_m) and an 8–10 amino acid peptide that is loaded into a binding groove on the HC. Assembly of class I molecules begins with the translocation of the HC into the endoplasmic reticulum (ER) where initial disulfide bond formation and domain folding occur with the assistance of the molecular chaperone calnexin and associated thiol oxidoreductase ERp57. This is followed by binding of the β_2_m subunit and incorporation of the HC-β_2_m heterodimer into a peptide loading complex (PLC) composed of calreticulin, ERp57, tapasin, and a peptide transporter termed TAP. Within the PLC, TAP translocates peptides from the cytosol into the ER lumen where the tapasin-ERp57 heterodimer exchanges low affinity peptides in the MHC class I binding groove for high affinity ones. Binding of a high affinity peptide is coupled to release of MHC I from the PLC and the ER, although there are additional quality control mechanisms that act following ER export [1,2]. Once on the cell surface, the peptide-MHC class I complex is scrutinized by the T cell receptors of CD8^+^ T cells. If recognized as foreign, as in the case of virus-derived peptides, the infected cell is killed.

Human cytomegalovirus (HCMV) is a beta herpesvirus that is prevalent throughout the human population. The virus produces a chronic, long-term infection that is due in part to its expression of numerous proteins that evade the CD8^+^ T cell arm of the adaptive immune system. These include US2, US3, US6, US10, and US11 [3,4] that use distinct mechanisms to interfere with the presentation of peptides by MHC I molecules. US3 retains MHC I within the ER but does not disrupt peptide loading [5], US6 binds to TAP and disrupts peptide translocation [6,7], US10 was originally thought to delay trafficking of MHC I [8] but was later shown to target HLA-G for degradation [9], and both US2 [10,11] and US11 [11,12] guide nascent MHC I heavy chains towards the ER-associated degradation (ERAD) pathway. ERAD is a host cell quality control process that removes misfolded proteins from the ER environment and its features differ depending on the location of the folding lesion [13,14]. Proteins with misfolded ER luminal regions can be directed to ERAD by various sensors that recognize misfolded segments directly (BiP, SEL1L) or by ERAD-specific lectins (OS-9, XTP3-B) that detect altered oligosaccharide structures. These sensors deliver the misfolded protein to retrotranslocation complexes via adaptors such as SEL1L that are associated with one of several E3 ligases, most commonly HRD1 for proteins with misfolded ER luminal segments. This is followed by retrotranslocation into the cytosol and degradation by the proteasome. Although the identity of the retrotranslocation pore remains unclear, the energy and mechanical force required for removal of the protein from the ER membrane is provided by the Cdc48/p97 AAA-ATPase complex in a process dependent on the addition of ubiquitin chains to the misfolded protein.

US2 and US11 are type I transmembrane proteins that localize to the ER and bind directly to MHC I through their ER luminal domains. They trigger rapid degradation of MHC I heavy chains early after their translocation into the ER. Both US2- and US11-mediated degradation mechanisms involve the molecular chaperone BiP [15] and the translocon-associated protein TRAM1 [16], and result in MHC I being ubiquitinated and retrotranslocated to the cytosol, de-glycosylated by peptide N-glycanase [17] and degraded by the proteasome. Despite these basic similarities, there are key differences in the ERAD machinery utilized by each immunoevasion molecule. US11 requires the ERAD machinery defined by the E2-recruitment protein AUP1, UBXD8 (ubiquitin regulatory X domain 8) [18], the E3 ligase TMEM129 [19,20], the E2 ubiquitin-conjugating enzyme Ube2J2 [19,20], and the polytopic membrane protein Derlin1 [21,22]. Although the ERAD adapter SEL1L [23] had previously been linked to US11 function, later work suggested that SEL1L may instead be involved in the degradation of US11 itself [24], US2 operates with a different set of cellular factors. The E3 ligase RNF139 (TRC8) [25] is required for US2 function and the thiol oxidoreductase PDI is thought to aid in the release of MHC I heavy chains from US2 prior to their degradation [26]. Additionally, signal peptide peptidase (SPP) has been shown to interact with PDI [26], US2 [27] and RNF139 [25] but conflicting SPP depletion studies raise questions concerning its functions in US2-mediated degradation of MHC I [27] [28]. Finally, the molecular chaperones calnexin and calreticulin have been shown to interact with MHC I in a US2-dependent manner although a functional contribution to MHC I degradation remains to be established [29].

How MHC class I is targeted to ERAD by US2 and US11 remains unclear. The crystal structure of US2 bound to MHC class I does not indicate any obvious unfolding or dissociation of the class I heterotrimer that might be recognized by various ERAD sensors [30]. Given that US2 and US11 can be co-isolated with various molecular chaperones and ERAD components [10,15,16,19,20,22,25,27] it seems likely that these immunoevasion molecules actively recruit MHC I to cellular ERAD pathways. Consistent with this view, MHC I degradation rates are much faster in US2- or US11-expressing cells [10,12] than those observed for misfolded MHC I in cells lacking these viral proteins [31]. Finally, US2 has been found to target other cellular and plasma membrane proteins for degradation, including integrin α-chains, CD112, IL12RB1, PTPRJ, and thrombomodulin, suggesting a mechanism of action that may be broader than originally appreciated [32].

Recently, a role was demonstrated for the ER luminal peptidyl prolyl isomerase cyclophilin B (CypB) in the degradation of soluble ERAD substrates [33]. In light of this, we investigated the potential role of ER-localized cyclophilins in MHC I ERAD induced by US2 and US11. We found that cyclophilin C (CypC), but not CypB, participates in US2-mediated degradation of MHC I and that neither cyclophilin was involved in degradation by US11. We hypothesized that CypC may promote complex formation between MHC class I, US2, and other ERAD components [34]. In investigating this model, we identified several ER proteins including malectin, PDIA6 (P5), and TMEM33 whose associations with US2 were altered upon CypC depletion or overexpression, and whose presence enhanced US2-mediated degradation of MHC I. These findings indicate that US2 co-opts a diverse array of host proteins to accomplish the efficient degradation of MHC I and subsequent evasion of immune surveillance by cytotoxic T cells.

## Experimental Procedures

### Cell culture and antibodies

All studies were conducted using the human glioblastoma astrocytoma cell line U373-MG as well as variants that stably express US2 or US11. These cells were provided by Dr. Hidde Ploegh (Whitehead Institute, MIT) and were maintained in high glucose DMEM (Life Technologies, Carlsbad, CA), supplemented with 10% fetal bovine serum, antibiotics (penicillin/streptomycin) and 2 mM glutamine.

The mouse monoclonal antibodies (mAb) HCA2 and HC10 were used to detect denatured MHC I heavy chains [35,36], and the mouse mAb W6/32 was used to detect folded (β_2_m-associated) MHC I [37]. Rabbit polyclonal antisera specific for the following proteins were used in this study: anti-calnexin generated as described previously [38], anti-cyclophilin C which cross-reacts with cyclophilins A and B (10287-2-AP, Proteintech Group, Chicago, IL), anti-cyclophilin B (ab16045, Abcam, Cambridge, UK) anti-PDIA6 (PA3008, Affinity Bioreagents, Golden, CO), anti-malectin (PA5-21408, Thermo Scientific, Waltham, MA), anti-TMEM33 (SAB1102315, Sigma Aldrich, St Louis, MO), anti-US2 and anti-US11 provided by Dr. Domenico Tortorella (Mount Sinai School of Medicine, New York) and anti-human α_1_-antitrypsin (A0409, Sigma Aldrich, St Louis, MO). Mouse anti-GAPDH was obtained from Millipore (MAB374, Billerica, MA) and mouse anti-HA mAb 12CA5 was provided by Dr. Tania Watts (University of Toronto). Goat anti-mouse Alexa 647-conjugated antibody (A21235, Life Technologies) was used for secondary staining in flow cytometry.

### Plasmids and mutagenesis

Wild type US2 and US11 expression constructs in a retroviral vector and C-terminally HA tagged US2 and US11 constructs in pcDNA3.1 were provided by Dr. Domenico Tortorella (Mount Sinai School of Medicine, New York). A pcDNA3.1 plasmid containing cDNA encoding the NHK variant of α_1_-antitrypsin [39] was obtained from Dr. Richard Sifers (Baylor College of Medicine, Houston). The HA-CypC construct in pcDNA3.1+ consisted of wild type CypC with an HA tag inserted at the N-terminus following the signal sequence. An additional CypC construct lacking the HA tag was synthesized by Life Technologies and was designed to be resistant to siRNAs CypC-(i) and CypC-(ii) (see below). Each siRNA recognition site was modified with at least 6 synonymous mutations. Site directed mutagenesis was then used to create two additional mutations, either R89A using forward primer 5`-CGGTTATAAAG-GAAGCAAGTTTCATGCTGTCATCAAGGATTTCATGATT-3`and reverse primer 5`-AATCATGAAATCCTTGATGACAGCATGAAACTTGCTTCCTTTAT-AACCG-3`, or K123A using forward primer 5`-ACATTTCCAGATGAGAACTTCGCACT-GAAGCACTATGGCATTGGGT-3`and reverse primer 5`-ACCCAATGCCATAGTGCT-TCAGTGCGAAGTTCTCATCTGGAAATGTC-3`. These were based on similar mutations in CypB which abolished catalytic activity [33,40] or calnexin binding [41], respectively. The siRNA-resistant wild type CypC and two mutant constructs were subcloned into the plasmid pLVX-ZsGreen1, resulting in pLVX-CypC^WT^, pLVX-CypC^R89A^, and pLVX-CypC^K123A^. HLA-A68 with an N-terminal HA tag (HA-HLA-A68) [42] was provided by Dr. Kwangseog Ahn (Seoul National University) and subcloned into pLVX-tdTomato. A wild type PDIA6 construct lacking any epitope tag and wild type US2 construct with three HA tags added to the C-terminus (US2-3xHA) were synthesized by Life Technologies and subcloned into pcDNA3.1+/Zeo and pLVX-IRES-tdTomato, respectively. Lastly, GIPZ shRNA vectors containing either a nonsense shRNA (GIPZ nonsense shRNA) or shRNAs specific for CypC (GIPZ305215, GIPZ305217, GIPZ 305218, and GIPZ305219) were obtained from Open Biosystems (Huntsville, AL).

### RNA interference

Stealth siRNAs (Life Technologies) were used for all knockdowns. CypC-(i) siRNA (PPICHSS108319) specifically targeted cyclophilin C, CypC-(ii) siRNA (PPICHSS108320) targeted both CypC and CypB, and CypB siRNA (PPIBHSS106317) specifically targeted CypB. The Stealth siRNA specific to PDIA6 was the same as used previously (PDIA6HSS11509) [43]. The Stealth negative siRNA (medium GC content) was used as a negative control (12935–300, Life Technologies). For knockdowns of proteins identified by LC-MS/MS, two to three siRNAs were pooled at equimolar ratios. These were CCT8HSS116512, CCT8HSS173838, and CCTHSS173839 for CCT8; MLECHSS114679, MLECHSS114680, and MLECHSS114681 for malectin; TMEM33HSS124009, TMEM33HSS124011, and TMEM33HSS1182893 for TMEM33; and RPN1HSS109348 and RPN1HSS184417 for Rpn1.

Knockdowns were carried out as described previously [43]. Briefly, 4 0μl of 20 μM siRNA was mixed with 490 μl of OPTI-MEM I and incubated 5 min at room temperature in each well of a 6-well plate. A 6 μl volume of Oligofectamine (Life Technologies) was added, mixed, and incubated 20 min at room temperature. Following the incubation 2.5 x 10^5^ cells in 1.5 ml DMEM + 2 mM glutamine were added, gently mixed, and cultured 4 h before addition of 1 mL DMEM + 2 mM glutamine + 30% FBS. For all knockdowns the procedure was repeated 3 days later to ensure complete knockdown of targets at the protein level.

### Lentiviral infection

Lentiviral production and infection protocols were based on those described by the RNAi Consortium (Broad Institute, MIT and Harvard University). For production, HEK293T cells were seeded in antibiotic-free growth medium and transfected with 1000 ng of the appropriate expression or shRNA lentiviral construct, 900 ng pCMV-PAX2, and 100 ng VSV-G-envelope plasmid pMD2.G using the TransIT-LT1 or Fugene6 transfection reagents. The cells were cultured for approximately 18 h following transfection followed by a medium replacement to high FBS medium (DMEM + glutamine + antibiotics + 30% FBS). Approximately 48 h later, the medium was harvested and filtered through a 0.22 μM syringe filter.

Lentiviral infections were carried out by diluting viral supernatant approximately 1/5 or 1/10 in normal DMEM medium and adding polybrene to a final concentration of 8 μg/ml. The diluted supernatant was added to the target cells, cultured for 24 h, and then the medium was replaced with normal DMEM. Infection efficiency was monitored by flow cytometry and detection of fluorescent markers (typically GFP, ZsGreen1, or TdTomato). Infection efficiencies greater than 95% were typically achieved.

### Quantitative PCR and Xbp-1 splicing assay

Detection of the unfolded protein response through Xbp-1 splicing was carried out as described previously [43]. Briefly, total RNA was isolated from cells using the RNeasy mini kit (Qiagen, Valencia, CA). Primers specific for both the spliced and unspliced forms of Xbp-1 mRNA (5`-GGAGTTAAGACAGCGCTTGG-3`and 5`-GAGATGTTCTGGAGGGGTGA-3`) were used in a one-step reverse transcriptase-PCR (Qiagen) to obtain fragments of differing sizes depending on the spliced state of the transcript.

For quantitative real-time PCR of mRNA levels, total RNA was first isolated using the Qiagen RNeasy Mini kit. The Life Technologies Superscript VILO cDNA Synthesis Kit with random primers was used to synthesize cDNA. Taqman real-time PCR using TaqMan Gene Expression Master Mix (Applied Biosystems) and pre-optimized TaqMan gene expression assays (PPIC (CypC)–Hs00181460, MLEC– 00207082_m1, TMEM33 –Hs01027901_m1, CCT8 –Hs00607229_mH, PDIA6 –Hs01012543_m1, HLA-A–Hs01058806_g1, RPN1 –Hs00161446_m1, and actin Hs01060665, Life Technologies) were used to quantify mRNA depletion and HLA-A transcript levels. In all cases, a 7500 Real-Time PCR System was used for analysis (Applied Biosytems) using the standardized protocol for these reagents. The delta delta threshold cycle (ΔΔCt) method was used to determine mRNA levels relative to the reference transcript beta actin [44]. Briefly, ΔΔCt = (Ct(target, untreated)–(Ct(reference, untreated))–(Ct(target, treated)–Ct(reference, treated)). This can then be expressed as 2^ΔΔCt^ to calculate the ratio of the target transcript to the reference transcript.

### Metabolic labeling and immunoisolation

U373-MG cells were starved of methionine and radiolabeled for 5–10 min with 100 μCi/ml of [^35^S] Met. Following radiolabeling, cells were either chased with 2 mM methionine-supplemented media or immediately prepared for lysis as follows. Cells were washed twice with chilled PBS and lysed in RIPA buffer (10 mM HEPES pH 7.4, 150 mM NaCl, 1% Nonidet P-40, 0.25% sodium deoxycholate, 0.1% SDS, 10 mM iodoacetamide, and 1x proteasome inhibitor cocktail (P8340, Sigma Aldrich)). The lysates were rocked for 30 min then centrifuged for 10 min at 11,000 rpm to remove nuclei and insoluble material. Supernatant fractions were treated with 10 μg w6/32, 10 μg HC10, and 10 μg HCA2 to isolate MHC class I and with 20 μl anti-calnexin antiserum as a radiolabeling and immunoisolation recovery control. After 2 h a mix of protein A-Sepharose (GE Healthcare) and protein G-Sepharose (Life Technologies) beads was added and incubated for 1 h. Beads were washed four times with 0.5% Nonidet P-40, 10 mM Hepes pH 7.4, 150 mM NaCl and then proteins were eluted, separated by SDS-PAGE under reducing conditions and visualized by fluorography. For inhibition of proteasomal activity, cells were pre-treated for 60 min with 25 μM lactacystin (Abcam), starved of methionine in the presence of 25 μM MG132 (Boston Biochem), radiolabeled in the presence of both inhibitors, and chased in the presence of 25 μM MG132.

### Flow Cytometry and Cell Sorting

Cells were trypsinized and washed in PBS + 3% FBS + 0.01% sodium azide prior to staining with 1.5 μg/100 μl W6/32 mAb for 30 min on ice. The cell pellet was washed and stained for 20 min with 1 μg/100 μl goat anti-mouse Alexa647. The cell pellet was washed, fixed in 0.5% paraformaldehyde in PBS, and strained, before analysis on a BD FACSCalibur flow cytometer (Faculty of Medicine Flow Cytometry Facility, University of Toronto).

Cell sorting for fluorophore labeled cells was carried out on a BD FACSAria IIu (Faculty of Medicine Flow Cytometry Facility, University of Toronto). Cells were trypsinized, washed in PBS + 10 mM Hepes + 1% BSA + 1 mM EDTA, and strained before sorting.

### Mass Spectrometry

Mass spectrometry to identify bait protein interaction partners was based on a protocol developed by Christianson et al. [45]. Three cell lines were created for these experiments: 1) U373-MG + nonsense shRNA which expresses the pLVX-empty and GIPZ nonsense shRNA vectors and was used to detect non-specific proteins isolated by this protocol, 2) U373-MG + US2-3xHA which expresses US2 with a triple HA tag at the C-terminus, and 3) U373-MG + US2 + HA-HLA-A68 which expresses both untagged US2 and N-terminally HA-tagged HLA-A68. Furthermore, the U373-MG + US2-3xHA cells were infected with lentivirus expressing either a GIPZ nonsense shRNA vector (U373-MG + US2-3xHA + nonsense shRNA), a lentiviral pool of four shRNAs (GIPZ305215, GIPZ305217, GIPZ 305218, and GIPZ305219) to deplete CypC (U373-MG + US2-3xHA + CypC KD), or a construct for overexpression of CypC (U373-MG + US2-3xHA + CypC OE). The U373-MG + US2 + HA-HLA-A68 cells were infected only with lentivirus containing the nonsense knockdown shRNA vector (U373-MG + US2 + HA-HLA-A68 + nonsense shRNA).

Approximately 1 x 10^8^ cells were incubated for 8 h with 25 μM MG132 and then were lysed in 10 mM HEPES pH 7.4, 150 mM NaCl, 1% digitonin, 10 mM iodoacetamide, and 1x proteasome inhibitor cocktail (P8340, Sigma Aldrich). Nuclei and insoluble debris were removed by centrifugation and lysates were incubated for 2 h with anti-HA conjugated agarose beads (Thermo Fisher Scientific, Waltham, MA) to isolate the tagged bait protein and any associated prey proteins. Beads were washed 3 times with 10 mM HEPES pH 7.4, 150 mM NaCl, 0.2% digitonin and 3 times with 50 mM ammonium bicarbonate pH 7.4. To elute bound proteins, the beads were incubated overnight at 37°C with 50 mM ammonium bicarbonate, pH 7.4 and 0.1% Rapigest (Waters, Cat. No. 186001860), a mass spectrometry compatible detergent that is hydrolysed at low pH. Following the incubation, the eluate was removed and any remaining material was eluted from the beads by boiling 5 min with 0.1% Rapigest, 50 mM ammonium bicarbonate, pH 7.4. Samples were reduced, alkylated with iodoacetamide and digested overnight with sequencing grade chemically modified trypsin (V5111, Promega, Madison, WI). The pH was reduced to 2 by the addition of trifluoroacetic acid to a final concentration of 0.5%, hydrolyzing the Rapigest detergent during a 45 min incubation at 37°C. Samples were centrifuged and the aqueous fraction was collected (discarding the insoluble portion of the detergent). Samples were concentrated in a SpeedVac concentrator (Savant) and desalted with C_18_ ZipTips (Millipore) for analysis on an Orbitrap Elite LC-MS/MS (SPARC Biocentre, Toronto).

The Peaks 7 software package (Bioinformatics Solutions Inc., Waterloo, ON) was used to analyze mass spectrometry results [46]. Peaks Studio calculates a *p*-value for each peptide, representing the probability that a false identification has occurred. These *p*-values are then represented as a significance score (-10log_10_(*p*-value)) for ease of consideration. For the US2-3xHA immunoisolations, only peptides with a significance score greater than 20 (*p*-value less than 0.01) were considered for subsequent analysis. Furthermore, peptides that were detected in only a single immunoisolation sample were not included. Identified peptides were then matched to the most likely originating protein, again with a stringent significance score of 20, and with a minimum of 2 unique peptides contributing to the match. HA-HLA-A68 immunoisolations were analyzed in a similar way but using a significance score of 5.

With the high precision mass spectra available from an Orbitrap mass analyzer, peptide area derived from the ion current versus retention time plot (quantified area) is used by the Peaks Studio software to estimate relative abundance of identical peptides between samples. Multiple unique peptides are considered for each protein and are used to provide a semi-quantitative estimate of protein abundance. This approach has been validated in a variety of applications [47–49]. We obtained protein abundance data for US2-3xHA immunoisolations (each consisting of two biological replicates) from cells expressing normal, depleted or overexpressed levels of CypC. To remove contaminating proteins from these results, protein abundances obtained from the anti-HA immunoisolation of U373-MG + nonsense shRNA cells were subtracted from the three US2-3xHA immunoisolations. The same background subtraction was applied to protein abundances obtained from the HA-HLA-A68 immunoisolation. Finally, to account for variations in immunoisolation and sample preparation between US2-containing samples, all protein abundances were normalized to those of the US2 bait protein.

## Results

### CypC, but not CypB, plays a role in US2-mediated degradation of MHC class I

**Fig 1:**
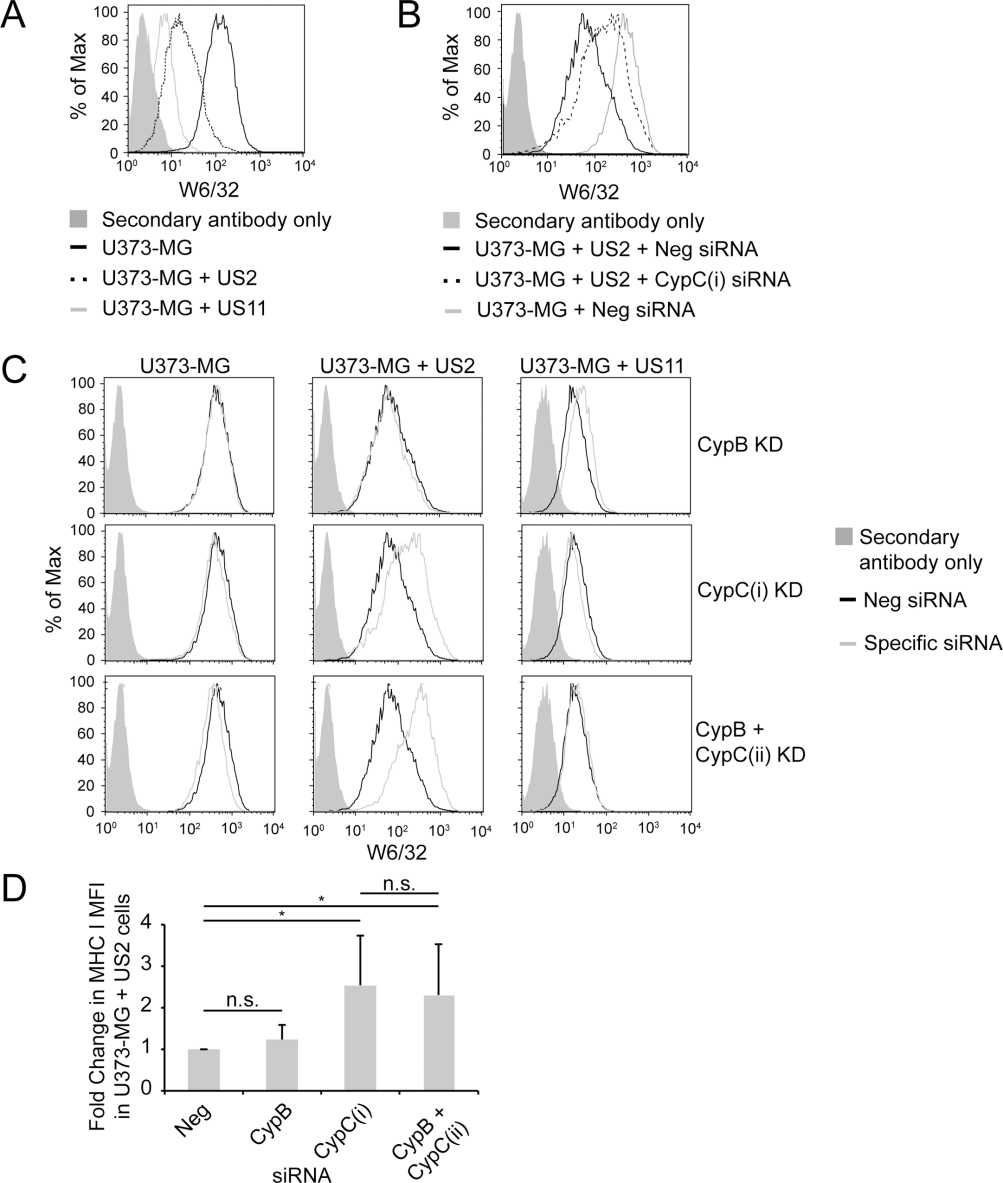
Depletion of CypC but not CypB increases MHC class I surface expression in US2-expressing cells. (A) U373-MG control cells or cells expressing US2 or US11 were stained with mAb W6/32 to detect surface MHC I molecules followed by goat anti-mouse Alexa647 secondary Ab and analyzed by flow cytometry. (B and C) U373-MG control, US2+, or US11+ cells were treated with CypB siRNA, CypC-(i) siRNA, or a cocktail of CypC-(ii) and CypB siRNAs mixed in a 3:1 ratio. Following treatments on day 0 and day 3, cells were replated on day 5 and analyzed by flow cytometry on day 6 (mAb W6/32). Data from panel B is included in and expanded on in panel C. (D) Average fold increase in surface MHC I (mAb W6/32) upon depletion of Cyp B (n = 8), Cyp C (n = 15), and CypB + CypC-(ii) (n = 8) in U373-MG + US2 cells. Student’s T-test was used to assess statistically significant differences from the Neg siRNA-treated cells, and between the CypC-(i) and CypB + CypC-(ii)-treated cells (* indicates *p-*value < 0.05; n.s., not significant). MFI = median fluorescence index

**Fig 2:**
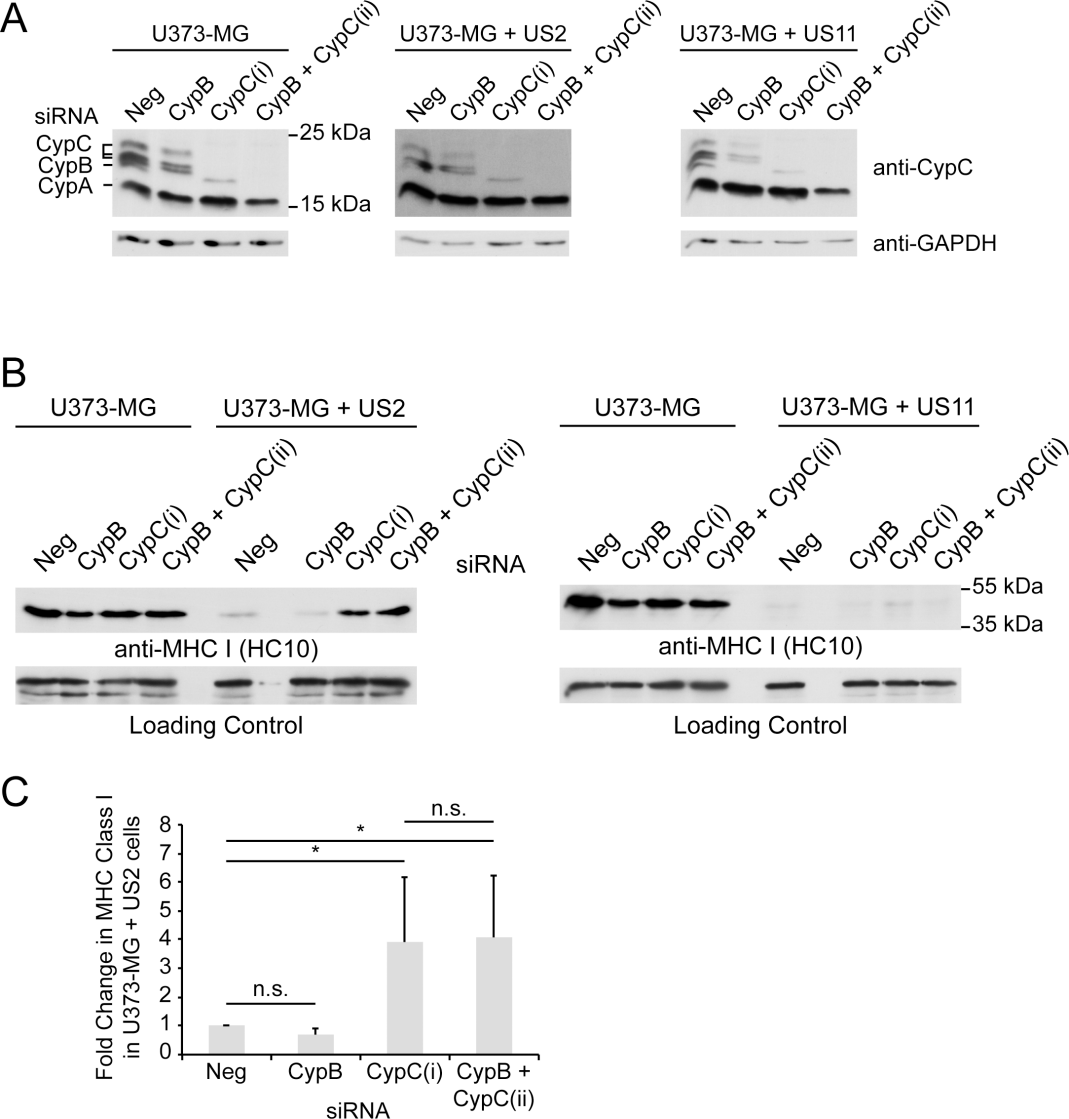
Depletion of CypC but not CypB increases total MHC class I in US2-expressing cells. (A) U373-MG control, US2+, or US11+ cells were treated with CypB siRNA, CypC-(i) siRNA, or a cocktail of CypB and CypC-(ii) siRNA. Cyclophilin depletion was evaluated on day 6 using anti-CypC antiserum which also detects CypA and CypB. Due to differing glycosylation states, CypC was typically observed as three distinct bands [50]. GAPDH was used as a loading control. (B) For the conditions described in panel A, MHC class I levels were assessed by immunoblot using mAb HC10; a representative background band was included as a loading control. (C) Average fold increase in MHC I in U373-MG + US2 cells upon depletion of Cyp B (n = 4), Cyp C (n = 6), and CypB + CypC-(ii) (n = 5). Student’s T-test was used to assess statistically significant differences from the Neg siRNA-treated cells, and between the CypC-(i) and CypB + CypC-(ii)-treated cells (* indicates *p-*value < 0.05; n.s., not significant).

Since previous work had demonstrated a role for CypB in ERAD [33], we were interested in determining if either of the ER-localized cyclophilins (CypB or CypC) was involved in MHC I ERAD mediated by US2 or US11. Human U373-MG cells stably expressing either US2 or US11 were used to examine the role of ER cyclophilins by RNA interference. As shown in Fig 1A, expression of either US2 or US11 resulted in the expected reduction of MHC class I at the cell surface. Depletion of CypC in US2-expressing cells increased surface levels of MHC I (Fig 1B). However, depletion of CypC in US11-expressing cells or in U373-MG control cells did not affect surface MHC I (Fig 1C). This effect was specific to CypC, since depletion of CypB had little effect on MHC I expression in any of these cell lines (Fig 1C). We also performed a combined knockdown using a mixture of CypB siRNA and an alternative CypC siRNA (that also targets CypB). Although there appeared to be a modest increase in surface MHC I compared to that obtained by depleting CypC alone (Fig 1C), this was not statistically significant in replicate experiments (Fig 1D). Similar results were obtained when total MHC I levels were analyzed by immunoblotting following efficient depletion of CypB, CypC or a combination of CypB and CypC (Fig 2A). An increase in MHC I was observed upon CypC depletion in US2+ cells but not in control or US11+ cell lines. (Fig 2B). Furthermore, the effect of combined depletion of CypB and CypC was not significantly different from CypC depletion alone (Fig 2B and quantified in Fig 2C). The specificity to US2 suggested that CypC was involved in US2-driven MHC I ERAD rather than participating in a more general pathway of MHC I homeostasis. In the latter case, CypC depletion would be expected to affect MHC I levels in U373-MG control or US11+ cells. Furthermore, this stabilization of MHC class I by CypC depletion was not due to loss of US2 protein expression since US2 protein levels remained unchanged after knockdown of CypC (S1A Fig)

Considering the potential for CypC to be involved in protein folding, we tested whether depletion of CypC was disrupting normal maturation of proteins within the ER. As an accumulation of misfolded proteins would be expected to induce an unfolded protein response, we used a PCR-based assay to monitor stress-induced mRNA splicing of the Xbp-1 transcription factor. No increase in spliced Xbp-1 mRNA was observed in our experiments (S1B Fig) which is consistent with our previous studies in human hepatoma cells [50]. In contrast, treatment of cells with cyclosporin A, an inhibitor of the catalytic activity of all cellular cyclophilins, did induce an unfolded protein response (S1B Fig) and hence cyclosporin A was not used in subsequent experiments.

### CypC depletion impedes the US2-mediated degradation of newly synthesized MHC I

**Fig 3:**
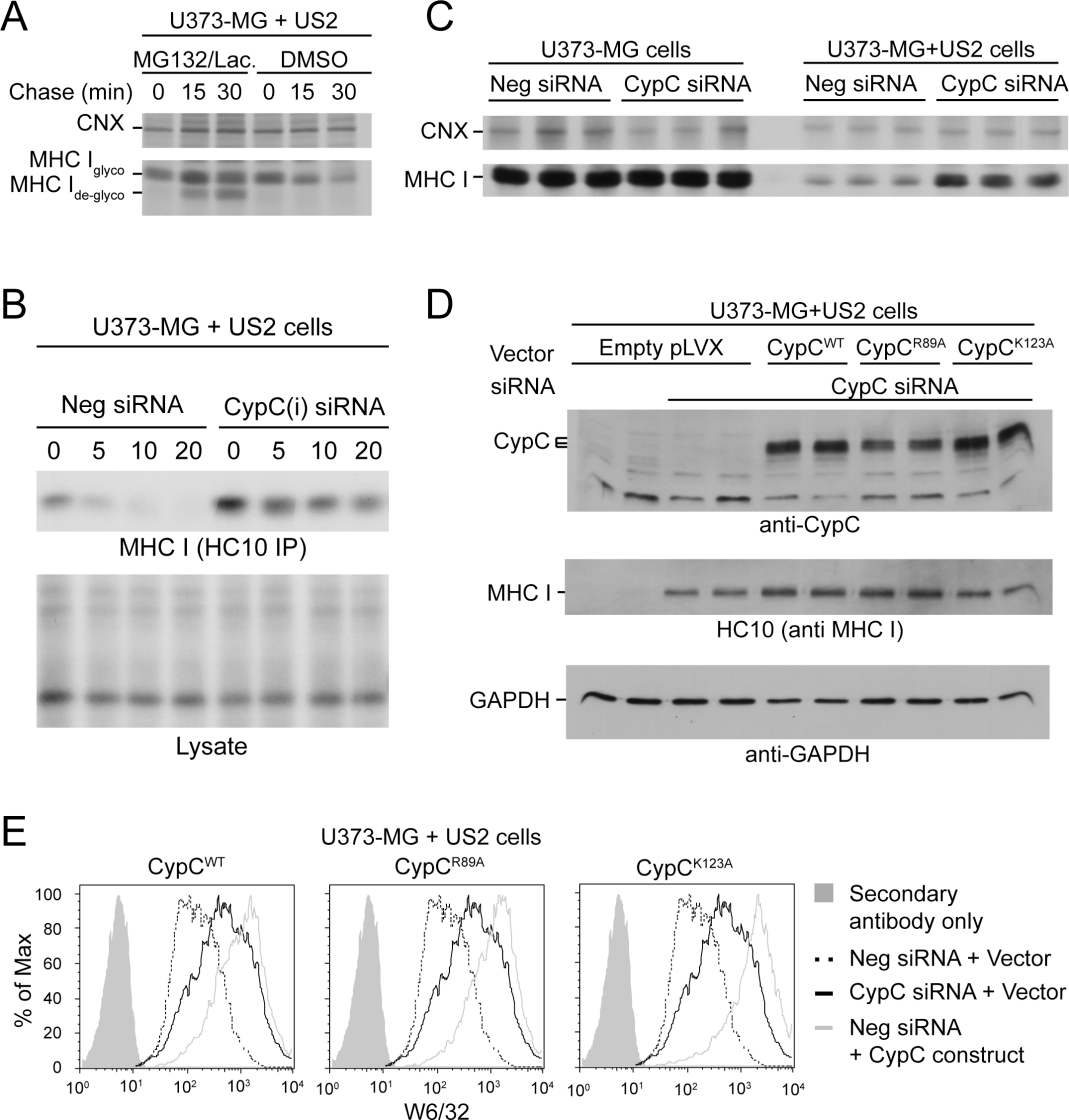
Both depletion and overexpression of CypC stabilize MHC I in cells expressing US2. (A) U373-MG + US2 cells were starved of methionine, radiolabeled with [^35^S]Met for 10 min and chased in Met-containing medium for the indicated times. Cells at each time point were treated either with DMSO in the starvation, pulse, and chase media, or with a combination of MG132 and lactacystin. Cells were lysed and MHC I was isolated using a cocktail of mAbs W6/32, HC10, and HCA2. Anti-calnexin was also included in the same immunoisolation to control for differences in radiolabeling and immunoisolation efficiencies. (B) U373-MG + US2 cells were treated with negative control or CypC-(i) siRNAs. On day 6 samples were radiolabeled for 5 min with [^35^S] Met. Cells were lysed following labeling or after chasing in Met-containing medium for the indicated times. MHC I was immunoisolated using mAb HC10. Cell lysate (lower panel) served as a control for differences in radiolabeling efficiency. (C) U373-MG and U373-MG+US2 cells were treated with negative control or CypC-(i) siRNAs and on day 6 triplicate samples were radiolabeled for 10 min with [^35^S] Met. Cells were lysed and MHC class I was immunoisolated as in panel A. (D) U373-MG cells were infected with lentiviruses prepared with either empty vector or vectors encoding siRNA-resistant forms of CypC^WT^, CypC^R89A^, or CypC^K123A^. Following infection, all cells were sorted for low expression of the plasmid marker (ZsGreen1). Cells were treated with CypC-(i) siRNA to deplete endogenous CypC and lysed on day 6. MHC class I levels were assessed by immunoblotting with mAb HC10. GAPDH served as a loading control. (E) U373-MG + US2 cells were infected with lentivirus prepared with empty vector and treated with either negative control or CypC-(i) siRNAs. Additional cells were infected with lentiviruses expressing either CypC^WT^, CypC^R89A^, or CypC^K123A^ and were treated with negative control siRNA. On day 6 following knockdown all cells were stained with mAb W6/32 for determination of surface MHC class I expression by flow cytometry.

US2 has previously been shown to down-regulate MHC class I through ERAD [10] and we wished to confirm that the increased MHC I levels observed with CypC depletion were consistent with impaired ERAD. As shown in Fig 3A, when US2+ cells were radiolabeled for 10 min and chased for periods up to 30 min, a rapid loss in newly synthesized MHC I heavy chain was observed. Consistent with ERAD disposal, this loss could be largely blocked by combined treatment with the proteasome inhibitors lactacystin and MG132. Proteasome inhibition also resulted in the accumulation of deglycosylated class I heavy chains that are normally degraded following retrotranslocation to the cytosol. To assess the effect of CypC depletion on this process, US2+ cells were subjected to RNA interference with negative control or CypC siRNAs, radiolabeled for 5 min and chased for up to 20 min. In cells depleted of CypC there was a striking increase in the amount of newly synthesized MHC I which was then degraded throughout the chase (Fig 3B). This suggested that CypC depletion mainly stabilizes MHC I during or shortly following synthesis. To confirm this, we radiolabeled both control and US2-expressing cells for 10 min without a subsequent chase and analyzed in triplicate. As shown in Fig 3C, comparison of the negative siRNA lanes in the two cell lines revealed that substantially less newly synthesized MHC I was recovered from the US2+ cells suggesting that in a 10 min pulse a substantial portion of MHC I was being degraded. Upon depletion of CypC, this degradation was impeded consistent with impairment in US2-mediated ERAD. Furthermore, in keeping with the previous immunoblotting experiments, CypC depletion had no effect on newly synthesized MHC I in control cells indicating the specificity of CypC in US2-mediated degradation as opposed to general effects on MHC I synthesis and folding processes within the ER (Fig 3C).

### Overexpression of CypC disrupts US2-mediated degradation of MHC class I molecules

To further validate the involvement of CypC in US2-mediated ERAD and to evaluate functional sites, we reintroduced siRNA-resistant versions of wild type CypC as well as functional site mutants into US2+ cells depleted of CypC. For functional site mutants, we tested R89A and K123A which correspond to mutations in CypB previously demonstrated to disrupt catalytic activity [33,40] and a calreticulin/calnexin-binding site [41], respectively. CypC has demonstrable peptidyl prolyl isomerase activity [51] but its ability to bind calreticulin or calnexin is less clear. Unexpectedly, overexpression of wild type CypC did not restore the rapid degradation of MHC I in US2+ cells depleted of CypC (Fig 3D, middle) which raised the possibility that the impaired MHC I degradation was due to an off-target effect of the CypC-directed siRNA. However, this was not the case as we found that simple overexpression of CypC without any knockdown caused a dramatic increase in MHC I levels as assessed by flow cytometry (Fig 3E, left). This suggested that US2-mediated degradation is sensitive to the expression level of CypC, and that both CypC depletion and overexpression impacts US2 function. Furthermore, overexpression of both CypC mutants caused a similar increase in MHC I expression (Fig 3D and 3E) indicating that the catalytic activity and potential interactions with lectin-chaperones are dispensable for the impaired MHC I degradation caused by CypC overexpression.

### CypC interacts with US2 and MHC class I

Since both overexpression and depletion produced similar phenotypes, we hypothesized that CypC may bridge the US2-MHC class I complex with ERAD machinery. In this scenario, depletion of CypC removes the bridge, whereas overexpression disrupts the stoichiometry, or ‘saturates,’ the link between US2 and ERAD components. Another possibility is that altered expression of CypC, as a component of a complex network of ER folding and quality control machineries [34], may indirectly impact US2 function by affecting the levels or spectrum of proteins associated with the US2-MHC I complex.

**Fig 4:**
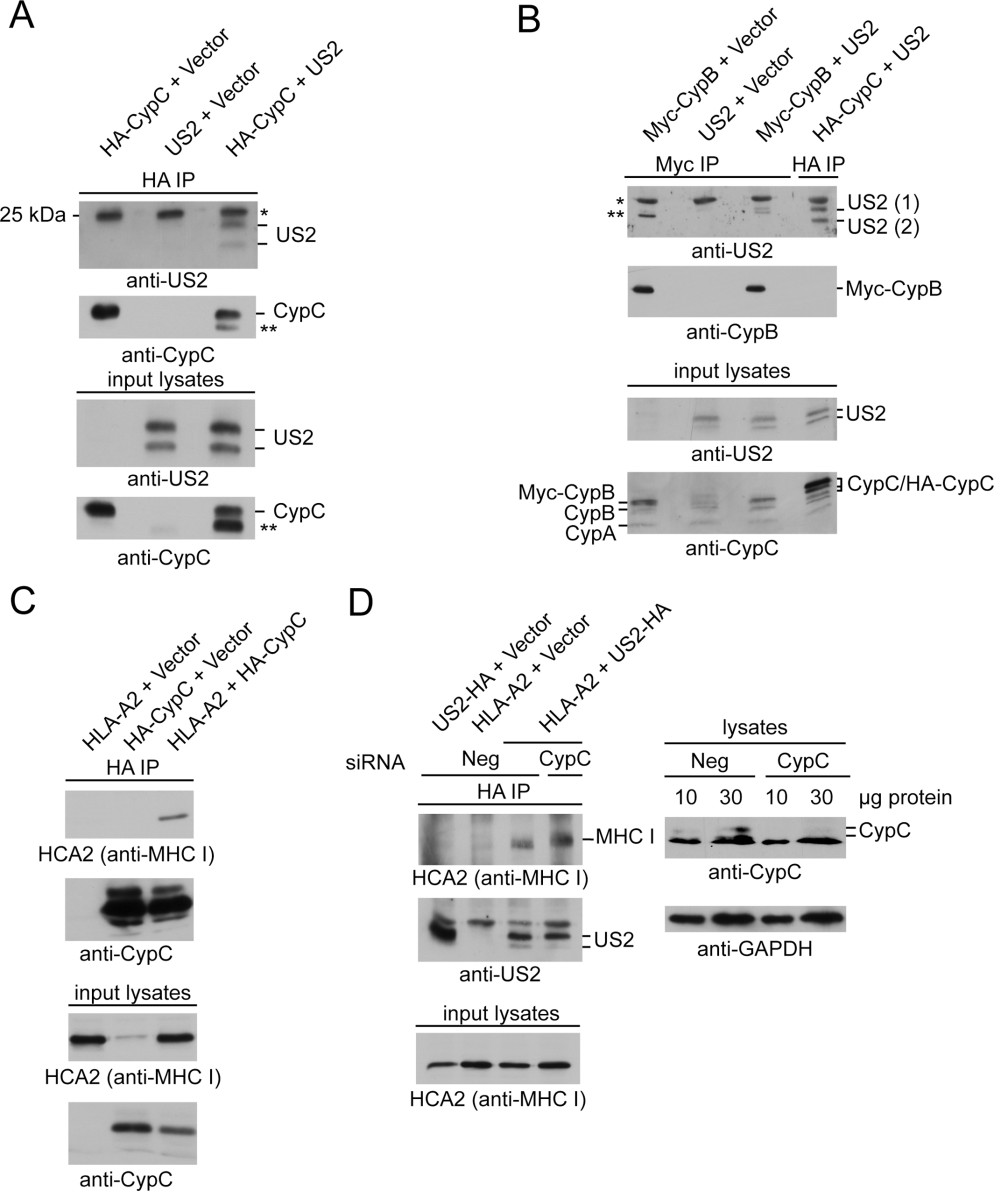
CypC co-isolates with US2 and MHC class I molecules. U373-MG cells were transiently transfected for 48 h with the indicated constructs in pcDNA3.1+/Zeo and then lysed in 1% digitonin lysis buffer followed by immunoisolation of epitope-tagged proteins. (A) CypC-US2 interaction. U373-MG cells expressing HA-CypC, US2, or HA-CypC + US2 were subjected to immunoisolation using anti-HA mAb and associated US2 was detected by immunoblot with anti-US2 antiserum. A background signal from the anti-HA mAb light chain is denoted by *. An unidentified band in anti-CypC immunoblots appearing below HA-CypC in cells transfected with both HA-CypC and US2 is denoted by **. (B) CypB does not interact with US2. U373-MG cells expressing myc-CypB, US2, myc-CypB + US2, or HA-CypC + US2 were lysed and immune complexes isolated with anti-myc or anti-HA mAbs as appropriate. US2 was detected by immunoblotting with anti-US2 antiserum. The myc-CypB construct produced two non-specific bands in an anti-US2 blot which are denoted by **, and can be observed most clearly in the Myc-CypB + Vector control lane. While these bands overlap with the upper US2 band (1), the lower species (2) can be clearly distinguished. The single * corresponds to the light chain of the anti-HA or anti-myc mAb. (C) CypC-HLA-A2 interaction. U373-MG cells transiently transfected with plasmids encoding HLA-A2, HA-CypC, or HLA-A2 + HA-CypC were subjected to immunoisolation with anti-HA mAb. Co-isolated HLA-A2 was detected by immunoblot with mAb HCA2. (D) Depletion of CypC does not disrupt the US2-MHC I interaction. U373-MG cells were treated with negative control or CypC-(i) siRNAs on day 0 and again on day 3. Cells were then transfected on day 4 with plasmids encoding US2-HA, HLA-A2, or US2-HA + HLA-A2. Immunoisolations of US2-HA were conducted on day 6 using anti-HA mAb and associated HLA-A2 was identified using mAb HCA2. CypC depletion was detected using anti-CypC antiserum with GAPDH as a loading control.

In either scenario, CypC might be expected to be present in a complex with US2 and MHC I. Indeed, co-expression of HA-tagged CypC with US2 resulted in robust co-immunoisolation of US2 with anti-HA mAb (Fig 4A). In contrast, when a myc-tagged CypB construct was expressed under similar conditions, no co-immunoisolation of US2 could be detected (Fig 4B), consistent with the lack of phenotype associated with CypB knockdown (Figs 1B and 2B). In addition, overexpressed HLA-A2 could be co-immunoisolated with HA-CypC (Fig 4C). Since this was observed in U373-MG cells (not expressing US2), it suggests that CypC is able to interact with MHC I under normal MHC I maturation conditions. Importantly, although CypC could be detected in association with both US2 and MHC I, it was not required for their interaction. As shown in Fig 4D, depletion of CypC did not impair the recovery of US2-MHC I complexes immunoisolated from cells transfected with both US2-HA and HLA-A2 (Fig 4D), Rather, there appeared to be an increased association of HLA-A2 with US2 consistent with reduced MHC I degradation in CypC-depleted cells.

### US2 and MHC class I interact with a diverse array of proteins

If CypC depletion/overexpression is modulating the quality control/ERAD machinery recruited by US2 to effect rapid MHC I disposal, one would expect to see alterations in the spectrum of proteins associated with US2 upon CypC depletion or overexpression. To this end, we used lentiviral infection to establish three stable cell lines for use in an unbiased LC-MS/MS proteomic screen. These included U373-MG cells co-expressing US2 with a triple HA tag along with either a nonsense shRNA, a mixture of four CypC-specific shRNAs to deplete CypC, or a construct that overexpresses CypC. We also prepared two control cell lines: U373-MG cells infected with control lentiviral vectors (U373-MG + non-sense shRNA) to identify proteins that interact non-specifically with anti-HA conjugated beads, and U373-MG expressing both US2 and HA-tagged HLA-A68. This latter control was used to filter hits from the US2-3xHA pulldown for those also present in an MHC class I pulldown, reasoning that such proteins might be more likely to play a role in US2-mediated degradation of MHC I.

All cell lines were incubated for 8 h with MG132 to inhibit MHC I degradation and to stabilize associated proteins, and then were lysed in digitonin, subjected to immunoisolation using immobilized anti-HA mAb, and recovered proteins were digested with trypsin and subjected to LC-MS/MS. The results were analyzed using the Peaks 7 software package [46] which provides a semi-quantitative estimate of protein abundance (see Methods). By normalizing protein abundances to the US2-3xHA bait protein in the various samples, the fold change in abundance of US2-associated proteins between samples with different CypC levels (endogenous, depleted and overexpressed) could be calculated. This data is shown in S1 Table for 177 identified prey proteins. Similarly, we identified 59 proteins in the HA-HLA-A68 isolations by LC-MS/MS and their relative abundances are shown in S2 Table.

**Fig 5:**
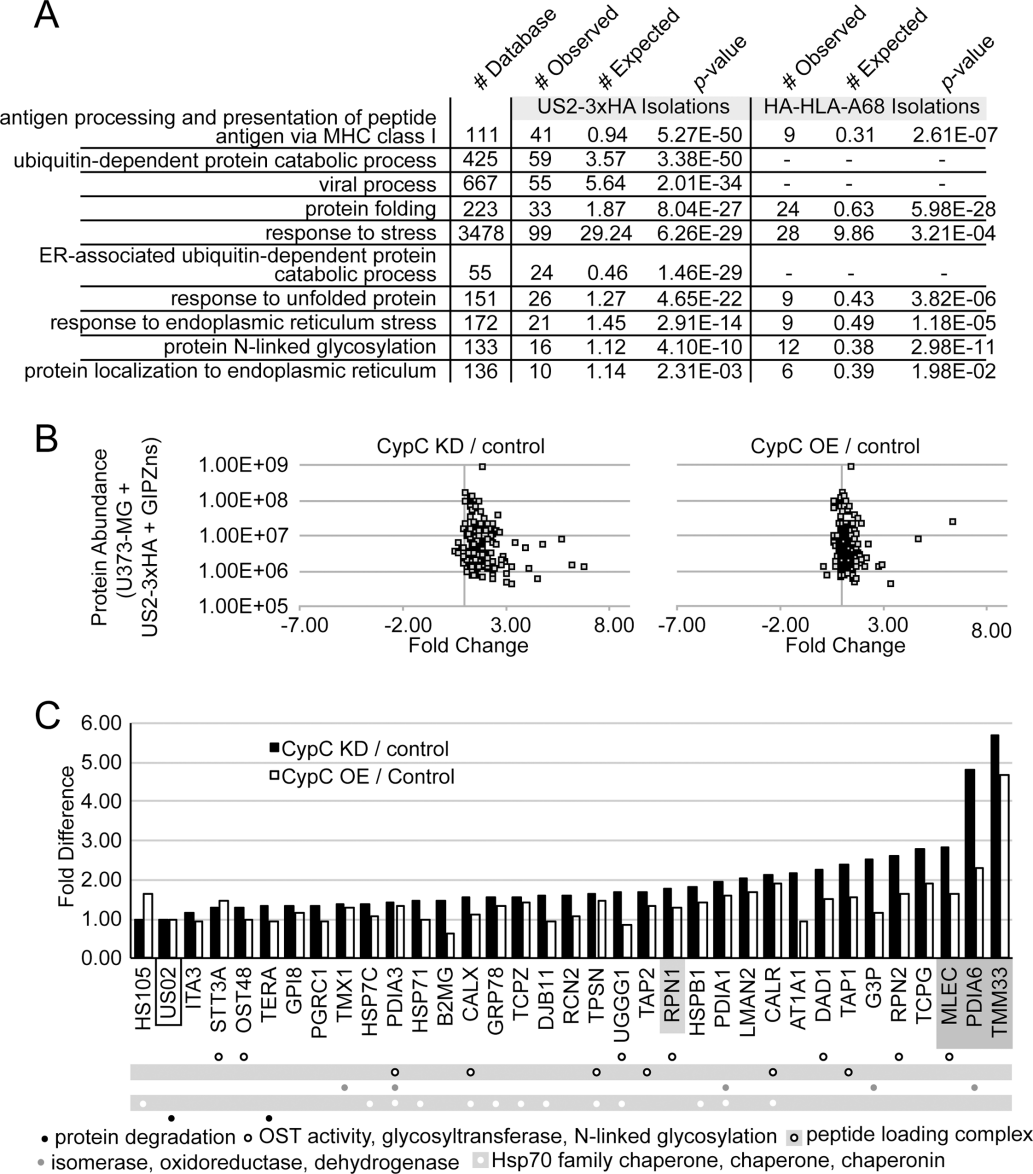
Identification of US2-associated proteins that are affected by CypC depletion or overexpression. (A) Gene Ontology classification of US2-3xHA- and HA-HLA-A68-interacting proteins. The frequency of proteins associated with particular GO terms in the LC-MS/MS datasets (# Observed, out of 177 identified proteins for US2-3xHA and 59 proteins for HA-HLA-A68) was compared to the number of proteins with that particular GO term in the GO Ontology database (# Database, out of 20,814 entries) to determine the frequency that a particular GO term would appear in the LC-MS/MS dataset by random chance (# Expected). The *p*-value indicates the probability that the # Observed for each GO term appears in the LC-MS/MS dataset by random chance. All *p*-values use the Bonferonni correction for multiple testing. (B) Many US2 interacting proteins change in abundance upon CypC depletion or overexpression. Quantified abundances of the various proteins detected in U373-MG + US2-3xHA isolations and their fold enrichment or depletion following CypC depletion (left panel) or overexpression (right panel) were plotted to evaluate overall changes. HNRPQ (SYNCRIP), CLH1 (CLTC), NEST (NES), CMC2 (SLC25A13), and THY1 are not plotted, as they are located outside the axis limits (S1 Table). Of the 174 identified proteins examined, 164 increased in association with US2-3xHA following CypC depletion and 135 increased in association following CypC overexpression. (C) Proteins detected in association with US2-3xHA were filtered against those detected in association with HA-HLA-A68 to include results common to each. The fold change in their abundance following depletion (black bars) or overexpression (white bars) of CypC is shown. Identification of selected GO terms is indicated below each set of bars as coded dots. US2 (which was used for normalization) is boxed, and hits selected for further validation are shaded in grey.

The results from the combined US2-3xHA immunoisolations and the HA-HLA-68 immunoisolation were analyzed using the Gene Ontology Consortium [52] and Panther Classification System [53] analysis tools. Identified proteins were examined for statistical enrichment of particular GO terms when compared to a database of 20,814 GO annotated proteins. By comparing the number of occurrences of a particular GO term in the LC-MS/MS results (#Observed) to the number of occurrences in the database (# Database), the likelihood of seeing a particular term by random chance in the LC-MS/MS dataset could be calculated (# Expected). A number of GO terms were enriched for proteins recovered in the US2-3xHA and HA-HLA-A68 isolations (Fig 5A compare #Observed and # Expected; S3 and S4 Tables). These included terms for protein folding, MHC I antigen presentation, cellular responses to stress, protein glycosylation, and endoplasmic reticulum localization of proteins. In addition, the US2-3xHA immunoisolations had a statistically significant number of proteins involved in ERAD and viral processes.

As an alternative means to visualize the types of proteins present in each LC-MS/MS dataset, we used STRING to identify relationships between these proteins [54]. By analyzing US2-3xHA-and HA-HLA-A68-associated proteins for experimental, database, text mining, and homology links, clusters of proteins tied to related functions or complexes could be identified. As expected, clusters related to ER-associated degradation and the peptide loading complex were present in both the US2-3xHA (S2 Fig) and HA-HLA-A68 (S3 Fig) datasets. Note that not all proteins identified by LC-MS/MS were displayed due to the high stringency required for links. Additionally, the US2-3xHA dataset contained a concentration of proteasome-related proteins. Surprisingly, a cluster of oligosaccharyl transferase (OST) complex and TRiC chaperone complex proteins were observed in the STRING analysis. While the presence of individual subunits could be present by chance, the representation of these complexes in both datasets suggested a potential role in US2-mediated degradation.

When the quantitative data from the US2-3xHA immunoisolations was compared between samples from cells expressing normal levels of CypC versus those depleted of CypC, a large number of hits increased in association with US2 upon CypC depletion rather than disappearing (Fig 5B, left panel). A similar trend was observed for CypC overexpression (Fig 5B, right panel). To focus the analysis of US2-interacting proteins to those most relevant to MHC I degradation, a list was constructed of proteins that were recovered in common from both the US-3xHA and HA-HLA-A68 isolations. The fold change in abundance of these hits was then examined following CypC depletion or overexpression (Fig 5C). A number of proteins involved in protein folding, such as molecular chaperones that may be involved in early targeting of MHC I for ERAD, as well as redox factors (isomerases, oxidoreductases) that could help unfold MHC I prior to retrotranslocation, were increased in association with US2 following knockdown or overexpression of CypC. Several of these, such as BiP, calnexin, calreticulin and the thiol oxidoreductase PDI, have previously been linked to US2 function [15,26,29]. In addition, we observed a large number of OST complex subunits as well as cytosolic TRiC chaperonin complex subunits (TCPG and TCPZ) that had also been identified in the STRING analysis.

The increase in the association of these proteins with US2 was intriguing, since we expected with the bridging model to see a reduction in associated ERAD/quality control components upon CypC depletion or overexpression. Rather, the overall increase in levels of these hits combined with the involvement of many of them in protein maturation processes suggested that modulation of CypC levels may ‘stall’ US2 in association with its interaction partners. In such a scenario, depletion of these interaction partners might also affect US2-mediated degradation of MHC I.

### Identification of TMEM33, PDIA6, and malectin as novel participants in US2-mediated degradation of MHC I

To test whether the proteins that were enriched in association with US2 following alteration of CypC expression were indeed playing a functional role in MHC class I degradation, we selected a number of hits for further analysis. TMEM33 was the top hit that was enriched upon CypC depletion or overexpression (Fig 5C). This predicted three-pass transmembrane protein is ER localized and is potentially involved in the regulation of reticulon proteins [55]. It is also induced upon ER stress and its overexpression modulates the activities of the unfolded protein response sensors IRE1 and PERK [56]. PDIA6, also known as P5 [57], was another prominent hit and as a thiol oxidoreductase could potentially be involved in reducing MHC I disulfides prior to retrotranslocation and degradation [58]. Malectin was also of interest, as this lectin has been linked to retention of misfolded proteins within the ER [59,60]. The oligosaccharyltransferase (OST) subunit ribophorin I (RpnI) was also selected due to its published interactions with malectin [61,62] and the fact that another OST subunit, ribophorin 2 (Rpn2), also exhibited increased association with US2 (Fig 5C). Lastly, the TRiC chaperone complex was targeted. Multiple subunits of this complex were detected in the US2-3xHA and HA-HLA-A68 immunoisolations (S2 and S3 Figs). One of the subunits, TCPQ (or CCT8), was chosen to examine potential involvement of the complex in US2 function. Although the identification of this chaperone complex may be artifactual due to its cytoplasmic localization and ability to interact with non-native substrates, the fact that multiple subunits were represented in our results bore further investigation.

**Fig 6:**
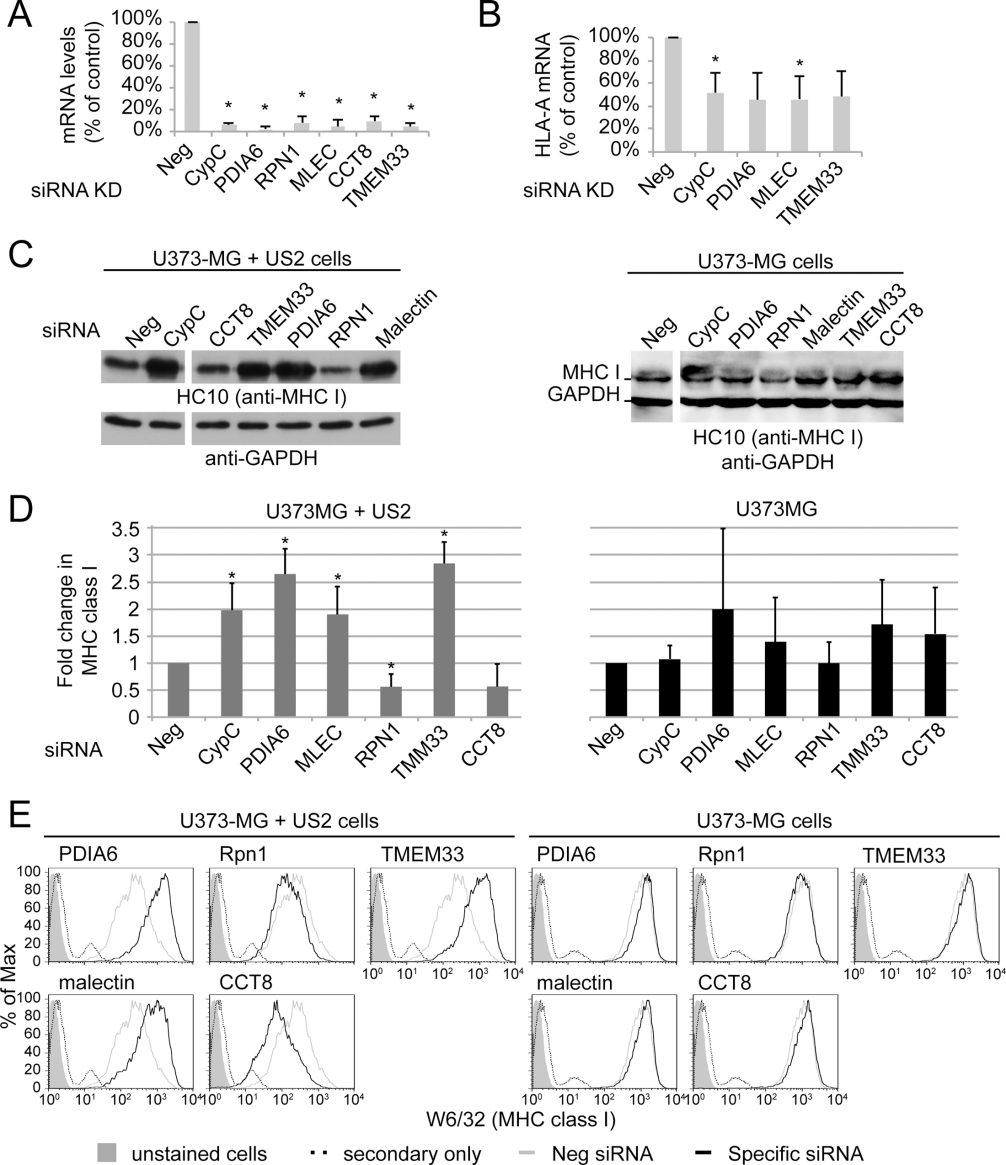
US2-associated proteins identified by modulating CypC expression participate in US2 degradative functions. (A) Determination of siRNA knockdown efficiency. The indicated targets of interest were depleted in U373-MG + US2 cells using 2–3 individual siRNAs transfected together on day 0 and boosted on day 3. On day 6 cells were lysed and RNA isolated for real-time PCR analysis. All knockdowns resulted in 90% or greater depletion of target mRNAs. Results were normalized to beta actin using the delta delta Ct method for comparison of knockdown treatments. An unpaired Student’s T-test was used to show statistically significant differences from the negative control siRNA (* indicates *p-*value < 0.05, n = 5). (B) Using a similar method, HLA-A transcripts were quantified under various knockdown conditions. (C) U373-MG + US2 cells and U373-MG control cells were treated with the indicated siRNA pools and on day 6 MHC class I levels were determined by immunoblotting with mAb HC10. (D) Quantified MHC I band intensities from the experiment in panel C. All results were normalized to the negative control siRNA sample for each cell line (* indicates *p*-value < 0.05, n = 4). (E) U373-MG + US2 cells and U373-MG control cells were subjected to knockdown as in panel (C) and surface MHC I levels were determined by flow cytometry following staining with mAb W6/32.

**Fig 7:**
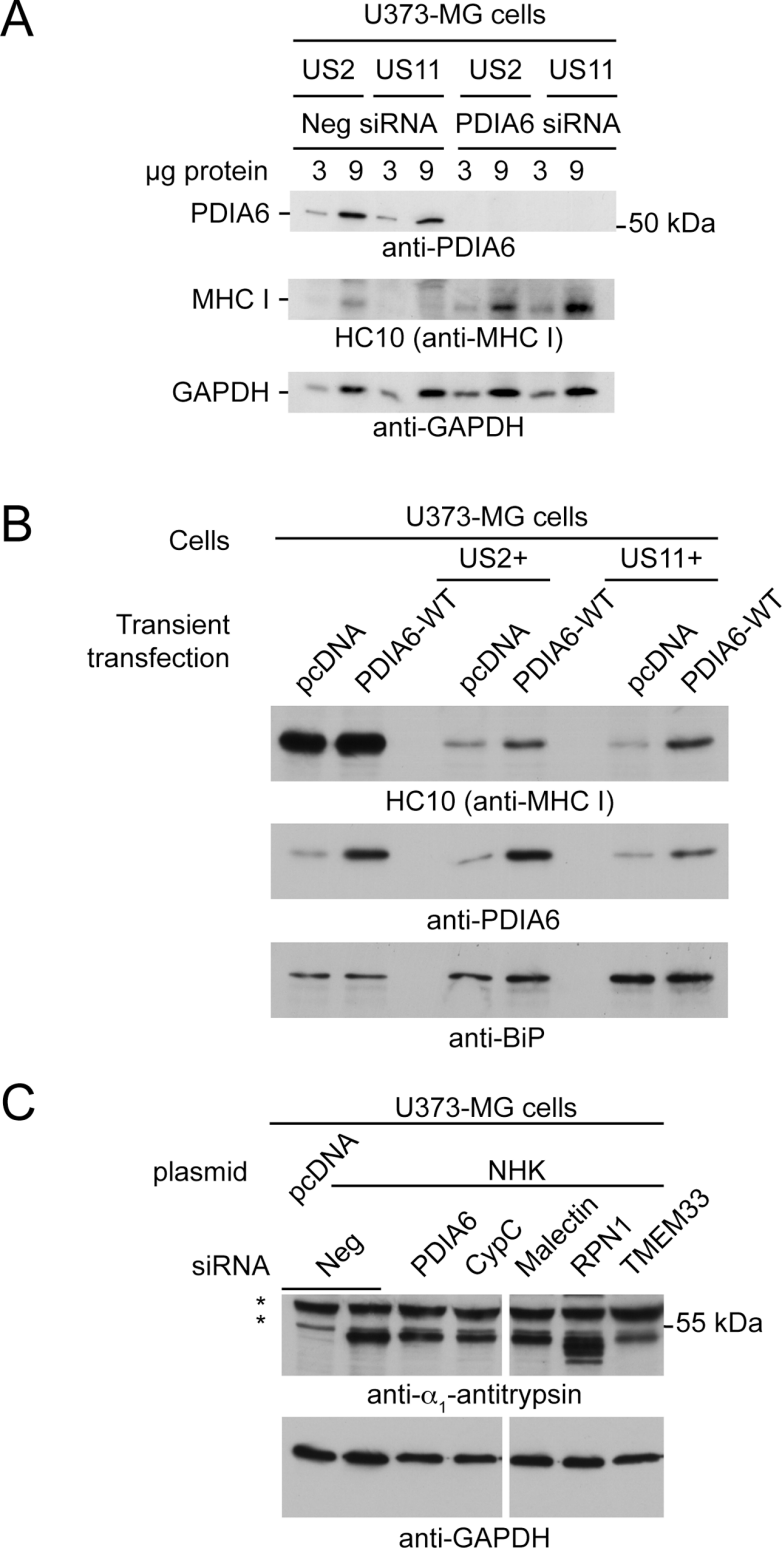
Involvement of novel US2-associated proteins in other ERAD pathways (A) US11-mediated degradation. U373-MG + US2 or + US11 cells were treated on day 0 and on day 3 with an siRNA specific for PDIA6 or a negative control. On day 6 cells were lysed and MHC I levels were determined by immunoblotting with mAb HC10. GAPDH served as a loading control. (B) U373-MG, U373-MG + US2, or U373-MG + US11 cells were transiently transfected with either empty pcDNA vector or pcDNA + PDIA6^WT^. After 48 h, MHC I levels were determined by immunoblot; BiP served as a loading control. (C) U373-MG cells were treated with the indicated panel of pooled siRNAs on days 0 and 3. On day 4 cells were replated and transiently transfected with an α_1_-antitrypsin NHK variant expression plasmid. Approximately 48 h later cells were lysed and NHK levels were determined by immunoblotting.

We first confirmed by IP-western the associations between US2 and malectin, TMEM33 and PDIA6, as well as the increased association between US2 and either TMEM33 or malectin following CypC overexpression or depletion (S4A Fig and S4B Fig). To assess whether these or any of the other selected proteins influenced US2-mediated degradation of MHC I, two to three siRNAs for each target were pooled for use with U373-MG + US2 cells. For PDIA6, a previously validated siRNA used in studies of its ER quality control functions was used [43] and CypC siRNA was included as a positive control. In all cases, efficient knockdown was observed at the mRNA level as assessed by qPCR (Fig 6A). Immunoblotting to detect MHC class I protein in these US2+ siRNA-treated cells revealed significant increases in MHC I upon depletion of CypC as expected, but also upon depletion of PDIA6, TMEM33, and malectin (Fig 6C, quantified in Fig 6D). No increases were observed in the levels of HLA-A transcript for any of these depletions, suggesting that any observed increases in MHC I protein levels were not a consequence of increased MHC I transcription or altered transcript stability (Fig 6B). In contrast, depletions of RpnI or CCT8 were not associated with an increase in steady state MHC I protein levels. No statistically significant increase in MHC I expression was observed for any of the knockdowns in U373MG control cells, consistent with PDIA6, TMEM33 and malectin being specifically involved in US2-mediated degradation (Fig 6C and 6D). These effects of target depletion were confirmed by flow cytometry. As expected, depletion of TMEM33, PDIA6, or malectin increased surface MHC I in US2+ cells with no impact of any of these knockdowns on surface MHC I in U373-MG cells (Fig 6E). Further investigation into PDIA6 revealed that in addition to playing a role in US2+ degradation of MHC I, it also stabilized MHC class I molecules in U373-MG cells expressing US11 (Fig 7A). Remarkably, in a manner reminiscent of CypC, over-expression of PDIA6 also increased the level of MHC class I in US2+ cells (and also in US11+ cells, Fig 7B).

### CypC, malectin, PDIA6, and TMEM33 do not affect the degradation of a soluble ERAD substrate

Following the observation that PDIA6 participates in both US2- and US11-induced degradation of MHC I, it was of interest to determine whether any of the proteins that were identified might be more broadly involved in ERAD. Consequently, we assessed the effects of their depletion on the degradation of a misfolded soluble ERAD substrate, the null Hong Kong (NHK) variant of α_1_-antitrypsin that is degraded by the HRD1 branch of ERAD [63]. As shown in Fig 7C, depletion of PDIA6, CypC, malectin, and TMEM33 had no effect on steady state NHK levels suggesting that they do not participate in ERAD of soluble glycoprotein substrates. In contrast, depletion of Rpn1 was associated with the appearance of more rapidly migrating forms of NHK, which were determined to be underglycosylated species by endo H digestion (data not shown), and there was also an increase in the total amount of NHK present. This may be due to impaired N-linked glycosylation by the OST complex, of which Rpn1 is a subunit, thereby affecting NHK degradation which is known to proceed through a glycan-dependent ERAD pathway [39]. In this context it is somewhat surprising that we did not observe a similar effect of Rpn1 depletion on the glycosylation state of MHC I (Fig 6C).

## Discussion

Cyclophilins have been implicated in the folding and functions of diverse proteins in several cellular compartments [64,65]. In the case of the ER, most studies have focused on CypB which is required for rhodopsin export from the ER in *Drosophila* photoreceptor cells [66] and for normal surface expression of the Na^+^-dicarboxylate cotransporter in HEK293 cells [67]. Furthermore, CypB utilizes its peptidyl prolyl isomerase activity in conjunction with the Hsp70 chaperone BiP to accelerate the folding of the IgG C_H_1 domain [68]. More recently, CypB has been implicated in ERAD since its depletion delayed the degradation of *cis*-Pro-containing soluble misfolded proteins [33]. In contrast, little is known about the only other ER-residing cyclophilin, CypC. We previously showed that although combined depletion of CypB and CypC had quite modest effects on the biogenesis of several *cis*-Pro-containing proteins in human hepatoma cells, these cells exhibited markedly increased ratios of oxidized:total glutathione suggesting cyclophilin involvement in the regulation of ER redox homeostasis [50]. In the present study, we uncovered the involvement of CypC in US2-mediated ERAD of MHC class I molecules. Both US2 and US11 trigger the degradation of MHC class I, but each utilizes a different subset of host cell proteins to accomplish this [18–21,23,25–27,29,69]. Consistent with this pattern, we found that CypC depletion impaired US2-mediated degradation but did not impact the mechanistically different immunoevasion molecule US11. This did not appear to be a general function of ER cyclophilins, since depletion of CypB did not affect MHC I degradation in US2-expressing cells.

Given that a previous study had implicated CypB-catalyzed isomerization of a *cis*-proline residue within a misfolded soluble substrate as being important for its disposal by ERAD, we considered whether either US2 or MHC I might be a substrate for the catalytic activity of CypC. We first examined the crystal structure of the ER luminal domain of US2 which revealed no *cis-*Pro residues, rendering it an unlikely substrate for CypC [30]. In contrast, the MHC I heavy chain does possess a single *cis*-proline within its conserved α3 domain [70–72]. We then attempted to address this issue directly by assessing whether wild type or catalytically inactive CypC could rescue the effects of CypC depletion. We found that overexpression of catalytically active CypC had a similar inhibitory effect on US2 function as did the depletion of CypC, and overexpression of a catalytically inactive form of CypC was just as effective as wild type CypC in impeding US2 function. Although these findings cannot address whether CypC catalytic activity is involved in US2 function at endogenous levels of expression, they do indicate that its activity is dispensable for its inhibitory effect upon overexpression. Given this finding and the sensitivity of US2 function to both over- and under-expression of CypC, we hypothesized that its effects on US2 function may be more likely to arise from the extensive network of interactions that ER peptidyl prolyl isomerases have with other components of the ER folding and quality control machinery [34].

To test this idea, we first confirmed by co-immunoisolation that CypC associates either directly or indirectly with both US2 and HLA-A molecules and that it is not required for the US2-HLA-A interaction. We then utilized a LC-MS/MS approach to characterize proteins associated with US2 and MHC class I, with the intent of detecting changes in these interactions upon CypC depletion or overexpression using label free quantification. This analysis revealed a surprisingly extensive network of US2-interacting proteins including well-established ERAD components and proteasome subunits, a variety of cytosolic and ER chaperones and co-chaperones, thiol oxidoreductases, components of the N-glycosylation machinery, and members of the MHC I peptide loading complex (Fig 5A; S2 Fig). When filtered for proteins also recovered when MHC I was isolated from US2-expressing cells, more than 30 proteins were detected (Fig 5C). However, upon CypC knockdown or overexpression, no consistent loss of any of these proteins could be observed. Instead, there was an increase in association of US2 with most of these proteins (Fig 5C). The basis for these increased associations remains unclear but it is conceivable that altered CypC levels influence the function or availability of one or more components of the US2-mediated degradation pathway. The resulting impaired US2 function might then cause it to accumulate with its interaction partners. Consistent with this notion, several of the proteins that increase in association with US2 upon altering CypC expression have previously been implicated in US2-mediated degradation of MHC I including BiP [15], calnexin, calreticulin [29], protein disulfide isomerase [26] and p97 (TERA) [73]. Consequently, we focused our attention on those proteins most strongly affected by CypC depletion and overexpression as a means to identify novel components of the US2 degradation machinery.

Using this strategy, we were able to validate through RNA interference the involvement of PDIA6, malectin and TMEM33 in US2-mediated degradation of MHC class I molecules. The involvement of the PDI family member PDIA6 was surprising given that PDI itself is known to participate in US2- (but not US11) mediated disposal of class I molecules [26]. However, it is the substrate binding function of PDI that is required in this process as opposed to its activity as a thiol oxidoreductase. It is generally thought that reduction of disulfides in misfolded substrates is a requirement for their disposal by ERAD and the PDI member ERdj5 has been shown to perform this function for some substrates [58,74]. Whether this is a requirement for ERAD of MHC I is unclear although agents such as diamide that promote disulfide formation do inhibit the degradation of MHC I by US2 and US11 [31]. For PDIA6, we showed that it is involved in both US2- and US11-mediated ERAD of MHC I, as well as having a potential effect on MHC I in U373-MG control cells. Therefore, PDIA6 may be exhibiting a broader effect on ERAD rather than an US2-specific role. Given that overexpression of PDIA6 had a similar inhibitory effect as knockdown, it seems unlikely that PDIA6 is playing a simple reductase role in unfolding MHC I for degradation. Future studies overexpressing catalytically inactive PDIA6 will be helpful in identifying the nature of its involvement as will be the determination of whether it acts directly on the MHC I substrate or on other components of the US2 and US11 degradation machinery.

We were also intrigued by the involvement of malectin in US2 function. This membrane-bound ER lectin with specificity for Glc_2_Man_9_GlcNAc_2_ oligosaccharide was initially thought to play a role in early glycoprotein biogenesis [75], but was later shown to delay secretion of misfolded proteins and, at least for one misfolded substrate, promote its degradation [59,60]. The apparent preference of malectin for misfolded proteins seems to be mediated through its association with Rpn1, a subunit of the oligosaccharyl transferase complex [61,62]. It has been suggested that the combined interaction of malectin and Rpn1 with the oligosaccharide and polypeptide segments of misfolded proteins, respectively, assists in the delivery of substrates to ERAD machinery [61]. Curiously, our depletion of Rpn1 did not show the same stabilization of MHC I in US2-expressing cells that we observed upon malectin knockdown. If malectin is indeed involved in the recruitment of MHC I for degradation, it is conceivable that in the presence of US2 the requirement for Rpn1 in substrate selection may be bypassed. Depletion of malectin did not stabilize the misfolded glycoprotein NHK, which is degraded via the HRD1 E3 ligase, indicating that malectin is not acting in all ERAD pathways. However, it remains to be determined whether malectin participates specifically in TRC8-mediated degradation or has a broader role in ERAD involving E3 ligases other than HRD1.

The final protein we implicated in US2 function is TMEM33 (also known as DB83), a predicted three-pass membrane protein localized to ER and nuclear membranes. Previous work has suggested a role for TMEM33 in regulating the ER tubule-shaping reticulon proteins [55]. Although it was the most highly enriched protein associated with US2 following CypC depletion or overexpression, and knockdown of TMEM33 impeded US2-mediated degradation of MHC I, the nature of its involvement in US2 function remains unclear. Large scale interaction studies have detected TMEM33 in association with VCP [76] and the deubiquitinating enzyme USP19 [77]. Both proteins have been linked to ERAD-related processes, with VCP promoting ERAD [78] and USP antagonizing it [79]. Furthermore, TMEM33 has recently been shown to be induced upon ER stress and to modulate the activities of the PERK and IRE1 stress sensors [56]. Thus TMEM33 has the potential to be a significant ERAD component acting on the TRC8 axis. As with malectin, TMEM33 did not participate in HRD1-mediated ERAD of NHK but a potential role in other ERAD pathways cannot be excluded. It will be of particular interest to assess its involvement in the disposal of other misfolded proteins and to extend the characterization of its interaction partners.

In summary, our findings suggest that US2 interacts with or is functionally associated with a much larger array of ER and cytosolic proteins than previously described [69]. The numerous and in some cases seemingly redundant proteins that contribute to efficient US2-mediated degradation support a model wherein US2 interacts with the complex quality control network that exists in the ER [34] rather than restricting itself to recruiting only select components involved in protein folding and degradation. Given the multitude of interactions that have been demonstrated between ER chaperones, co-chaperones, PDI family members and peptidyl prolyl isomerases [34] it is perhaps not surprising that depletion or overexpression of individual components such as CypC or PDIA6 may perturb this network with accompanying effects on US2 function. The multitude of US2 interacting proteins may also reflect the recent finding that the targets of this immunoevasion molecule are broader than previously appreciated, with US2 also targeting integrins and other plasma membrane proteins for degradation in addition to MHC class I [32].

## Supporting Information

S1 Fig Increased MHC I expression upon CypC depletion is not due to US2 instability or ER stress. (A) U373-MG cells were treated with CypC-(i) siRNA and US2 protein was monitored by immunoblotting on day 6 using anti-US2 antiserum. Calnexin and GAPDH were used as loading controls. CypC depletion and increased MHC I levels were verified by immunoblotting with anti-CypC antiserum and mAb HC10, respectively. (B) U373-MG + US2 cells were treated with CypB or CypC-(i) siRNA and RNA was isolated 6 days later. Primers specific to both spliced and unspliced Xbp1 mRNA were used to identify activation of the unfolded protein response in a one-step reverse transcriptase PCR. The PCR products corresponding to the unspliced and spliced forms are indicated. Cells were treated overnight with 20 μg/mL cyclosporine A as a positive control for Xbp1 splicing.
(TIF)

S2 Fig STRING analysis of proteins identified in US2-3xHA isolations. Proteins identified by LC-MS/MS from immunoisolations of US2-3xHA (S1 Table) were submitted for characterization using the STRING_10_ web software that displays protein relationships based on experimental evidence, database links, and homology links.
(TIF)

S3 Fig STRING analysis of proteins identified in HA-HLA-A68 isolations. Proteins identified by LC-MS/MS from immunoisolations of HA-HLA-A68 (S2 Table) were characterized in a similar manner to that described in S2 Fig
(TIF)

S4 Fig TMEM33, malectin, and PDIA6 co-isolate with US2. (A) US2-3xHA stably expressed in U373-MG cells or in cells with stable depletion (CypC KD) or overexpression (CypC OE) of CypC was immunoisolated from digitonin lysates using anti-HA mAb and immunoblotted for associated malectin and TMEM33. * denotes background bands present in immunoblots of cell lysates. ** denotes a background band observed in all IP samples following immunoblotting for TMEM33. (B) U373-MG cells transiently transfected with plasmids encoding US2, PDIA6-HA, or US2 + PDIA6-HA were subjected to immunoisolation with anti-HA conjugated beads. Co-isolated US2 was detected by immunoblot with polyclonal anti-US2 serum. Two different exposures of the US2 immunoblot are shown.
(TIF)

S1 Table Peaks Studio 7 data from LC-MS/MS analysis of US2-3xHA interacting proteins following background subtraction and normalization to bait protein. (XLSX)

S2 Table Peaks Studio 7 data from LC-MS/MS analysis of HA-HLA-A68 interacting proteins following background subtraction. (XLSX)

S3 Table Analysis of S1 Table results using the GO Ontology database and PANTHER Overrepresentation Test. (XLSX)

S4 Table Analysis of S2 Table results using the GO Ontology database and PANTHER Overrepresentation Test. (XLSX)

S5 Table Proteins identified in both US2-3xHA (S1 Table) and HA-HLA-A68 (S2 Table) LC-MS/MS analyses. (XLSX)
