# Supplementary figures and images for: Cyclophilin C Participates in the US2-Mediated Degradation of Major Histocompatibility Complex Class I Molecules
Source: PLoS One. 2015 Dec 21;10(12):e0145458. doi: 10.1371/journal.pone.0145458 (PMC4686535; doi:10.1371/journal.pone.0145458)

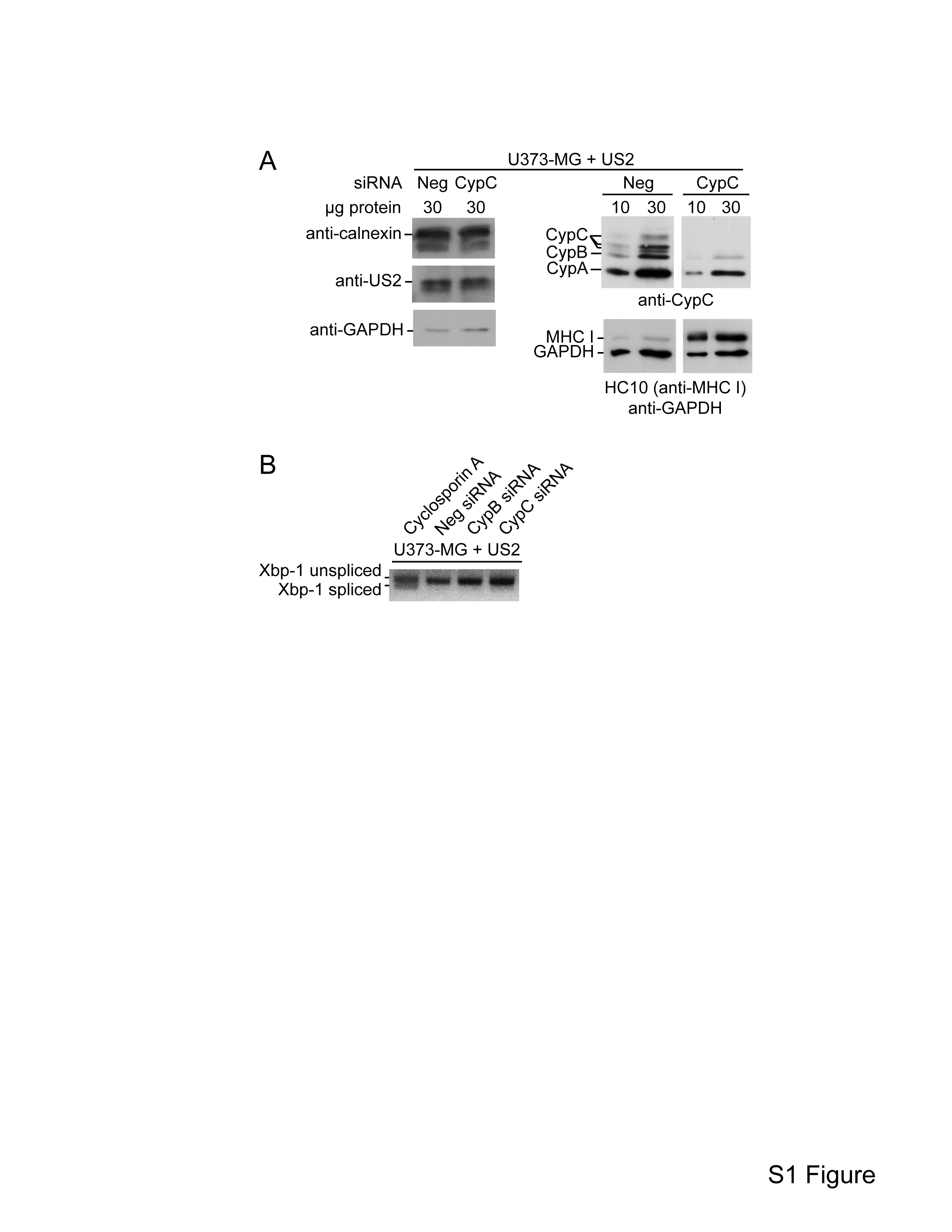

Supplement: S1 Fig — (A) U373-MG cells were treated with CypC-(i) siRNA and US2 protein was monitored by immunoblotting on day 6 using anti-US2 antiserum. Calnexin and GAPDH were used as loading controls. CypC depletion and increased MHC I levels were verified by immunoblotting with anti-CypC antiserum and mAb HC10, respectively. (B) U373-MG + US2 cells were treated with CypB or CypC-(i) siRNA and RNA was isolated 6 days later. Primers specific to both spliced and unspliced Xbp1 mRNA were used to identify activation of the unfolded protein response in a one-step reverse transcriptase PCR. The PCR products corresponding to the unspliced and spliced forms are indicated. Cells were treated overnight with 20 μg/mL cyclosporine A as a positive control for Xbp1 splicing. (TIF) [file pone.0145458.s001.tif]

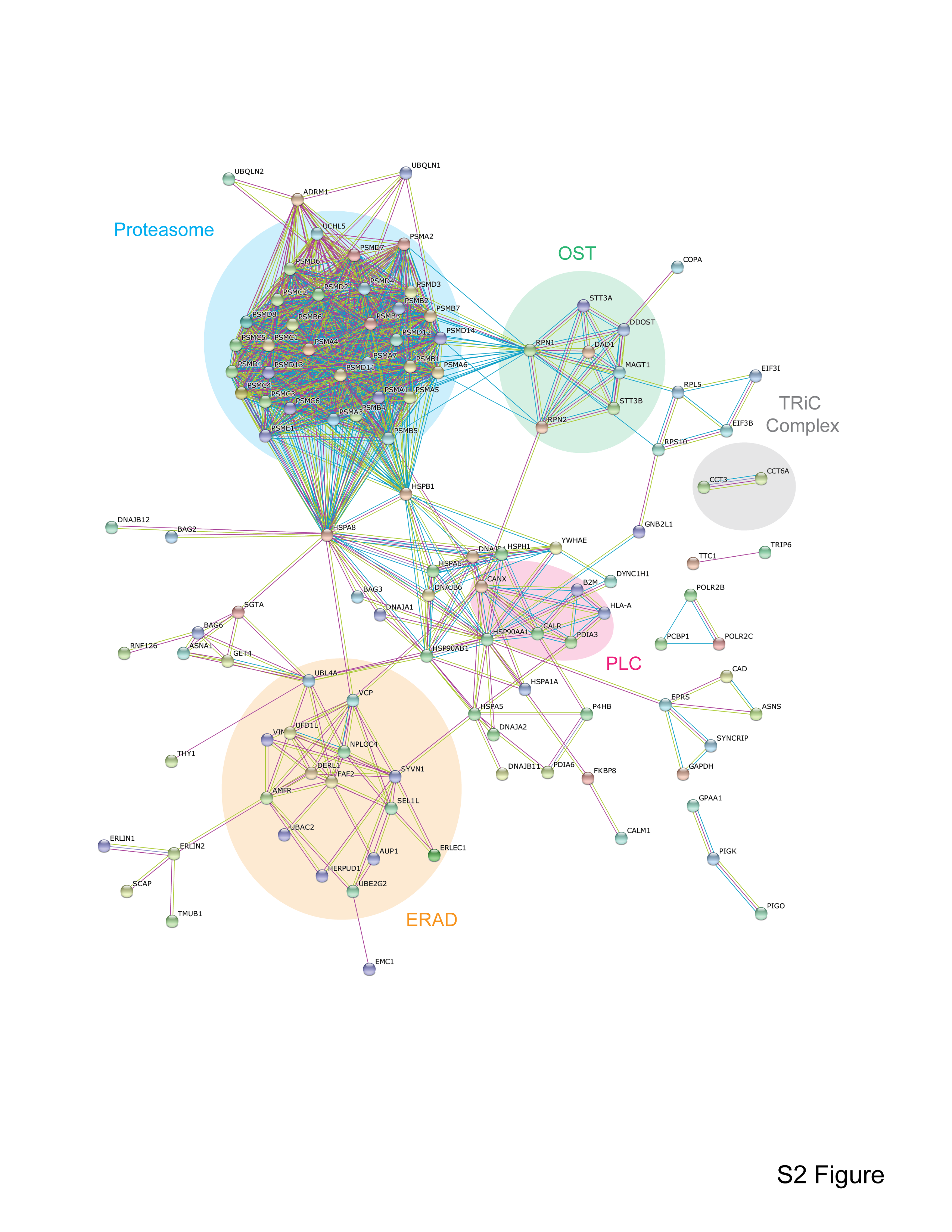

Supplement: S2 Fig — Proteins identified by LC-MS/MS from immunoisolations of US2-3xHA (S1 Table) were submitted for characterization using the STRING10 web software that displays protein relationships based on experimental evidence, database links, and homology links. (TIF) [file pone.0145458.s002.tif]

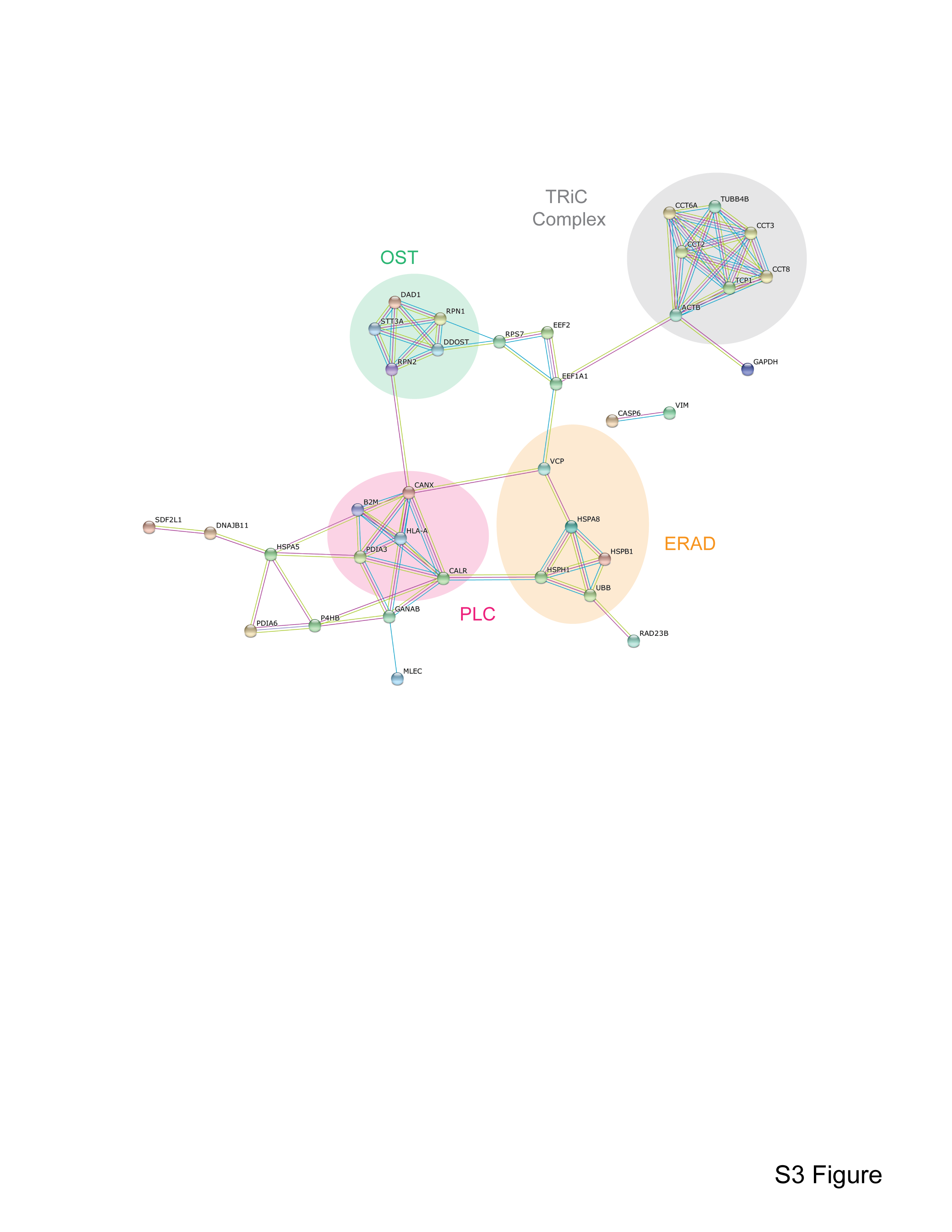

Supplement: S3 Fig — Proteins identified by LC-MS/MS from immunoisolations of HA-HLA-A68 (S2 Table) were characterized in a similar manner to that described in S2 Fig (TIF) [file pone.0145458.s003.tif]

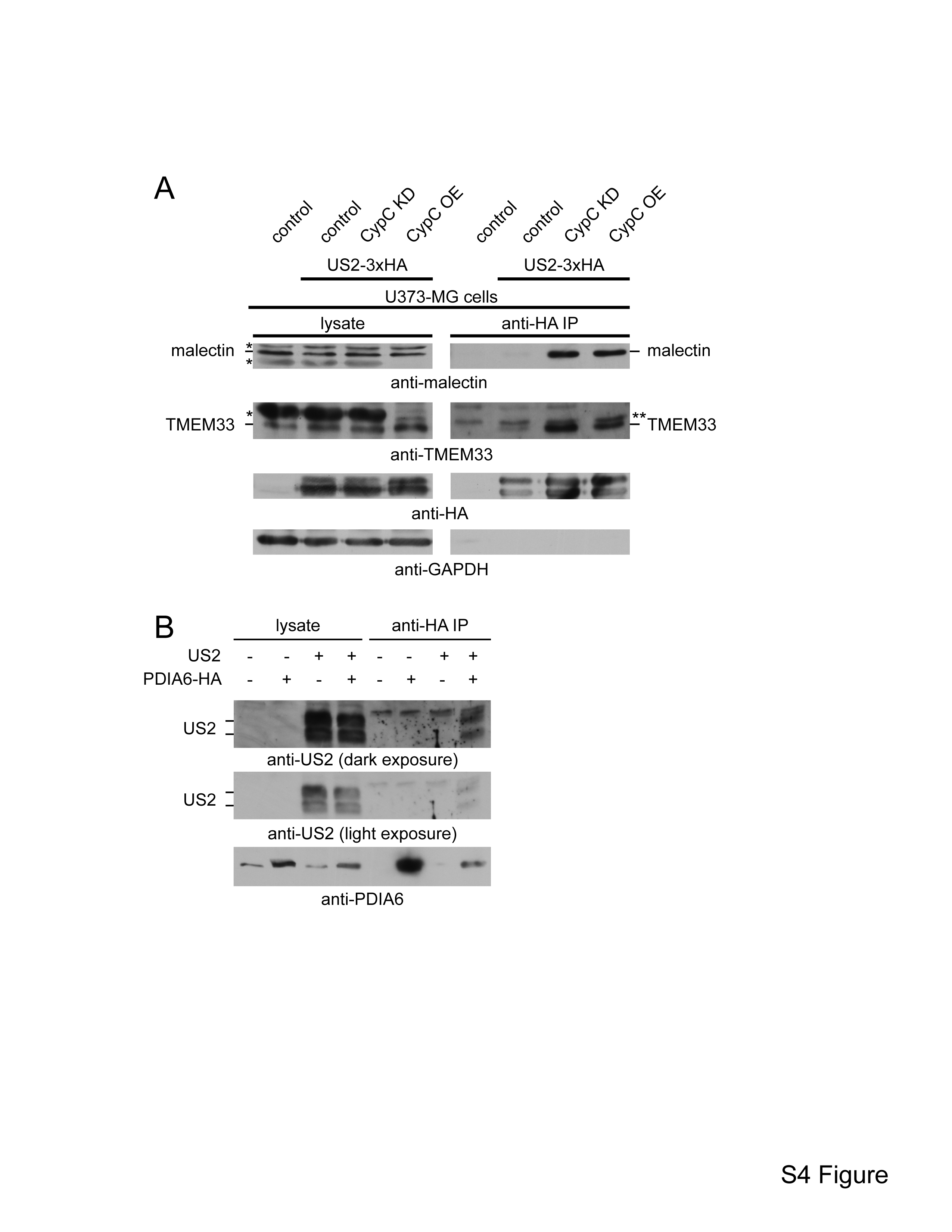

Supplement: S4 Fig — (A) US2-3xHA stably expressed in U373-MG cells or in cells with stable depletion (CypC KD) or overexpression (CypC OE) of CypC was immunoisolated from digitonin lysates using anti-HA mAb and immunoblotted for associated malectin and TMEM33. * denotes background bands present in immunoblots of cell lysates. ** denotes a background band observed in all IP samples following immunoblotting for TMEM33. (B) U373-MG cells transiently transfected with plasmids encoding US2, PDIA6-HA, or US2 + PDIA6-HA were subjected to immunoisolation with anti-HA conjugated beads. Co-isolated US2 was detected by immunoblot with polyclonal anti-US2 serum. Two different exposures of the US2 immunoblot are shown. (TIF) [file pone.0145458.s004.tif]
